# Ameliorative Effect of Quercetin against Abamectin-Induced Hemato-Biochemical Alterations and Hepatorenal Oxidative Damage in Nile Tilapia, *Oreochromis niloticus*

**DOI:** 10.3390/ani12233429

**Published:** 2022-12-05

**Authors:** Abdallah Tageldein Mansour, Heba H. Mahboub, Rehab M. Amen, Marwa A. El-Beltagy, Amany Ramah, Abdelfattah M. Abdelfattah, Hossam S. El-Beltagi, Tarek A. Shalaby, Hesham S. Ghazzawy, Khaled M. A. Ramadan, Adnan H. M. Alhajji, Heba S. Hamed

**Affiliations:** 1 Fish and Animal Production and Aquaculture Department, College of Agriculture and Food Sciences, King Faisal University, Al-Ahsa 31982, Saudi Arabia; 2 Fish and Animal Production Department, Faculty of Agriculture (Saba Basha), Alexandria University, Alexandria 21531, Egypt; 3 Department of Aquatic Animal Medicine, Faculty of Veterinary Medicine, Zagazig University, Zagazig 44511, Egypt; 4 Department of Zoology, Faculty of Science, Mansoura University, Mansoura 35516, Egypt; 5 Biochemistry Department, Faculty of Veterinary Medicine, Suez Canal University, Ismailia 41522, Egypt; 6 Graduate School of Medicine and Veterinary Medicine, University of Miyazaki, 1-1 Gakuen Kibanadai-nishi, Miyazaki 889-2192, Japan; 7 Department of Forensic Medicine and Toxicology, Faculty of Veterinary Medicine, Benha University, Qalyubia 13518, Egypt; 8 Department of Clinical Pathology, Faculty of Veterinary Medicine, University of Sadat City, Sadat City 32897, Egypt; 9 Agricultural Biotechnology Department, College of Agriculture and Food Sciences, King Faisal University, Al-Ahsa 31982, Saudi Arabia; 10 Biochemistry Department, Faculty of Agriculture, Cairo University, Giza 12613, Egypt; 11 Department of Arid Land Agriculture, College of Agricultural and Food Science, King Faisal University, Al-Ahsa 31982, Saudi Arabia; 12 Date Palm Research Center of Excellence, King Faisal University, Hofuf 31982, Saudi Arabia; 13 Central Laboratories, Department of Chemistry, King Faisal University, Al-Ahsa 31982, Saudi Arabia; 14 Department of Zoology, Faculty of Women for Arts, Science & Education, Ain Shams University, Cairo 11757, Egypt

**Keywords:** ivermectin, hematobiochemical, Nile tilapia, organ’s dysfunction, oxidative stress, quercetin

## Abstract

**Simple Summary:**

Aquatic pollution is an unavoidable danger with the spread use of agrochemicals in different agriculture sectors. Abamectin (ABM) has been one of the most widely used pesticides in recent decades due to its effectiveness in crop protection and pharmaceutical applications. The present study evaluated the effects of exposure to sublethal levels of ABM on several health and stress indicators of Nile tilapia, *Oreochromis niloticus,* and the potential protective effect of quercetin. The results showed that ABM exposure induced anemia, proteinemia, and hyperlipidemia in the serum of exposed fish and induced liver and kidney dysfunctions and oxidative damage. The dietary supplementation of quercetin ameliorates the negative effects of ABM on Nile tilapia’s physiological status and can be used as an antioxidant to mitigate the destructive effects of insecticide toxicity in aquaculture.

**Abstract:**

Abamectin (ABM) is a common agricultural pesticide and veterinary anthelmintic drug. It can discharge from the sites of application to aquatic systems via surface run-off or spray drift, causing harmful effects to aquatic organisms. The present study investigated the protective effect of dietary quercetin supplementation on hemato-biochemical parameters and hepato-renal oxidative stress biomarkers in Nile tilapia (*Oreochromis niloticus*) exposed to a sublethal dose of ABM. Fish were allocated into six equal groups. The first group was kept as a control group. The second and third groups (Q_400_, and Q_800_) were fed diets supplemented with two quercetin levels (400 and 800 mg/kg diet), respectively. The fourth group (ABM) was intoxicated with 20.73 µg/L of ABM. The fifth and sixth groups (ABM + Q_400_, and ABM + Q_800_) were fed diet supplemented with two quercetin levels (400 and 800 mg/kg diet) and simultaneously intoxicated with ABM for 60 days. The results showed that ABM significantly decreased RBCs, hemoglobin content, hematocrit, total protein, albumin levels, and acetylcholinesterase activity activities compared to the control. Meanwhile, ABM significantly increased white blood cells, glucose, total lipids, cholesterol, and alanine and aspartate aminotransferase activities. Liver and kidney levels of lipid peroxidation was significantly increased, while hepato-renal antioxidant biomarkers (reduced glutathione, super oxide dismutase, catalase, and total antioxidant capacity) were significantly decreased upon ABM exposure. On the other hand, quercetin dietary supplementation improved the hemato-biochemical alterations and alleviated oxidative stress induced by ABM exposure. Fish supplemented with quercetin at a level of 800 mg/kg diet showed better alleviating effects against ABM compared to 400 mg/kg diet. Based on these study findings, we suggest that quercetin dietary supplementation (800 mg/kg) offered direct protection against ABM-induced physiological disturbance and oxidative stress in Nile tilapia.

## 1. Introduction

Agrochemicals are widely used in the agricultural industry for pest and herb management, and for stimulating the production of different crops [1]. Abamectin (ABM) is one of the most frequently used pesticides in the last few decades, owing to its effectiveness in crop protection and pharmaceutical applications [2]. ABM is a macrocyclic lactone disaccharide compound, produced by natural fermentation of soil-dwelling actinomycete, *Streptomyces avermitilis* [3,4]. It is a mixture of avermectins, containing about 80% avermectin B1a and 20% avermectin B1b [5]. ABM is used as an insecticide, an acaricide [6], a nematicide [7], and as a multi-purpose antiparasitic substance in livestock [8]. It also has a slight toxic effect on earthworms and birds [6]. In addition, ABM has a wide use as an antiparasite in farm animals [8]. Accordingly, ABM can flow from the application sites and reach aquatic ecosystems, causing harmful effects to non-target fish and other aquatic organisms, even at low levels (μg/L) [9,10,11].

In fish, different responses from acute to chronic toxicity have been reported with ABM exposure. The reported adverse effects of ABM on fishes were behavioral changes, growth depression, hematological and biochemical disturbances, hepatorenal toxicity, immune depression, and oxidative stress, as reported in Zebra fish, *Danio rerio* [12], hybrid catfish, *Clarias macrocephalus* × *C. gariepinus* [13], Nile tilapia, *Oreochromis niloticus* [14,15], *O. mossambicus* [16], African catfish, *C. gariepinus* [17,18], and common carp, *Cyprince carpio* [19]. In addition, the exposure to chemical pollutants in aquatic animals induces hypergeneration of free radicals [20,21,22]. Reactive oxygen species (ROS) increases beyond the capacity of the cellular antioxidant defense system, leading to oxidative stress, causing injury to cell membranes, organelles, DNA, micronutrients, and inducing apoptosis [23,24,25]. Many investigators reviewed the induction of oxidative stress and depletion of antioxidant system components in different fish species exposed to different types of pesticides [26,27,28,29]. Accordingly, strengthening the antioxidant system in the animal body could alleviate the drastic effects of pesticides on animal physiological status.

Among powerful antioxidants in nature, quercetin is the major representative of the flavanol subclass of flavonoids, which is widely present in plants, fruits, vegetables, and multi-dietary supplements [30]. It has several beneficial properties, including scavenging ability of ROS, protecting lipids from peroxidation, and chelating metal ions [31,32]. As a result, quercetin has been used to treat a variety of disorders in both traditional and modern medicine, including cancer, infections, inflammation, oxidative stress, allergic reactions, and cardiovascular disease [33,34,35,36,37]. In aquaculture, dietary quercetin supplementation improved the growth performance of Nile tilapia [38], blunt snout bream, *Megalobrama amblycephala* [39] and snakehead fish, *Channa argus* [40]. It also revealed a lipid lowering effect in the serum and whole fish body of Nile tilapia [38], triglycerides, and cholesterol in blunt snout bream [39]. In addition, dietary quercetin reduced lipid peroxidation (LPO) and improved antioxidant status in vital tissues of silver catfish and blunt snout bream [39,41]. Quercetin showed an immunostimulant activity by improving non-specific immune response biomarkers and regulating immune-related gene expression in snakehead fish [40] and common carp [42]. However, there is no available studies regarding the use of quercetin against pesticide exposure in Nile tilapia and aquatic animal in general. For that, the aim of the present study was to evaluate the potential protective effects of quercetin against the destructive effects of ABM on the hematological, biochemical profiles, hepatorenal functions, acetylcholine level, and antioxidant status of Nile tilapia.

## 2. Materials and Methods

### 2.1. Chemicals

Abamectin (Vertimec 1.8% EC) was purchased from Syngenta Co., Basel, Switzerland. Its nomenclature, 5-O-demethyl avermectin A1a (i) mixture with 5-O-demethyl-25-de (1-methyl propyl-25-(1-methyl ethyl) avermectin A1a (ii). Quercetin (content > 98%, purchased from Nanjing Zelang Medical Technology Co., Ltd., Nanjing, China). All other biochemical kits were purchased from Bio-Diagnostic Co., Cairo, Egypt.

### 2.2. Experimental Fish

A total number of apparent healthy 270 Nile tilapia fingerlings were obtained from Abbassa farm for aquaculture and fisheries, Egypt, with an average body weight (20.00 ± 0.50 g). Fish were transferred to the laboratory and kept in identical glass aquaria containing dechlorinated tap water supported with air pumps under laboratory circumstances (dissolved oxygen 6.00 ± 0.7 mg/L, alkalinity 120 mg/L, hardness 153 mg/L CaCO_3_, pH 7 ± 0.5, temperature 26–27, and photoperiod 12:12 light: dark). The fish was fed on a basal diet for two weeks for acclimatization. The daily water exchange level was 50%.

All procedures of the experiment were conducted under the ethical guidelines and approved by the Institutional Animal Care and Use Committee (IACUC), Faculty of Veterinary Medicine, University of Sadat City, Egypt (Ethical approval number: VUsc-008-1-22).

### 2.3. Experimental Design

Fish were divided into six equal groups each with three replicates (15 fish in each replicate) and kept in glass aquaria 85 L of dechlorinated water. The first group of fish was kept as a control, while the second and third groups were fed a diet supplemented with quercetin (400 and 800 mg/kg diet: Q_400_ and Q_800_, respectively). The fourth group was exposed to ABM (20.73 µg/L: ABM); this dose was 1/10 of LC_50_, as determined by [43] and [15], who estimated LC_50_ as 207.360 µg/L. The fifth and sixth groups were exposed to ABM (20.73 µg/L) and fed a diet supplemented with quercetin at a dose of 400 and 800 mg/kg diet, ABM + Q_400_ and ABM + Q_800_, respectively. The selected doses of quercetin were based on the recommendation of [38]. The experiment was continued for 60 days, during which the water exchange rate was 50% with tap water, except for the group exposed to ABM, which was supplied with water with the same level of ABM.

### 2.4. Experimental Diet

The experimental diet ingredients and proximate chemical compositions were presented in Table 1. Briefly, the ingredients were purchased from the local market, ground and mixed well, and the quercetin was added according to the respect doses (0, 400, and 800 mg/kg). The diets were moistened with 300 mL water/kg diet at 45 °C, then pelleted using a meat mincer. The resulting pellets (3 mL) were dried and stored at −4 °C until used.

The proximate chemical composition was conducted according to AOAC [44]. Briefly, dry matter was determined by drying the samples to a constant weight at 105 °C in a drying oven (GCA, model 18EM, Precision Scientific Group, Chicago, IL, USA). The crude protein was determined by Kjeldahl distillation unit (UDK 129, VELP Scientifica, Usmate Velate, Italy). The crude lipids were evaluated using Soxhlet extractor glassware with petroleum ether (60–70 °C) for 10 consecutive cycles. Muffle furnaces (Barnstead/thermolyne Benchtop 47,900, Thermo Scientific, Waltham, MA, USA) were used to measure the ash content. The crude fiber was determined by acid and alkaline digestions method.

### 2.5. Blood Sampling

At the end of the experiment, ten fish from each group were collected and anesthetized with a solution of 0.02% benzocaine. Blood samples were obtained from the caudal fish vessels and divided into two parts. The first part was collected using sodium citrate as an anticoagulant for hematological parameters determination. In the second section, the collected blood samples were allowed to coagulate at room temperature in centrifuge tubes, and then centrifuged for 15 min at 5000× *g* at 4 °C. The obtained sera stored at −20 °C to be used for the determination of the biochemical parameters.

#### 2.5.1. Hematological Examination

The total count of red blood cells (RBCs) and white blood cells (WBCs) were determined by a hemocytometer and Schwa’s solution as diluent [45]. Hemoglobin (Hb) values were determined using cyanomethemoglobin method according to Collier [46]. The hematocrit value was estimated by microhematocrit tubes and centrifuge at 5000× *g* for about 5 min. as described by Wintrobe [47]. Blood Indices, including mean corpuscular hemoglobin (MCH), mean corpuscular hemoglobin concentration (MCHC), and mean corpuscular volume (MCV), were assessed according to the method of Gupta [48] and following formulas:

MCH (pg/cell) = 10 × [Hb (g/100 mL blood)/RBCs (10^6^/mm^3^)] (1)

MCHC = 100 × [Hb (g/100 mL blood)/hematocrit (%)](2)

MCV(fL) = 10 × [hematocrit (%)/RBCs (10^6^/mm^3^] (3)

#### 2.5.2. Biochemical Examination

All biochemicals parameters were determined using commercial kits of Bio-diagnostic Co., Cairo, Egypt. Serum Alanine (ALT) and aspartate (AST) aminotransferase activities were determined calorimetrically according to Reitman [49]. Glucose was evaluated using the method described by Young [50]. Serum total protein and albumin levels were measured using the methods described by Henry [51] and Doumas, et al. [52], respectively. Serum globulin was determined by subtracting albumin from the total sample protein. The serum total cholesterol level was measured using the enzymatic colorimetric method of Allain, et al. [53]. The total lipids were measured using the methods of Frings, et al. [54]. Serum creatinine and urea were estimated according to [55] and [56], respectively. Serum activity of acetylcholinesterase activity (AChE) was measured using the colorimetric kinetic method of [57].

### 2.6. Tissue Sampling and Antioxidant Status

The same anathemized fish that used for blood collection were dissected and liver and kidney tissues were collected. Tissue samples were homogenized in cold phosphate buffer saline (0.1 M pH 7.4) using a Potter-Elvehjem glass/Teflon homogenizer. After filtration and centrifugation at 1600× *g* for 10 min at 4 °C, the supernatant was obtained and stored at −20 °C, until oxidative stress biomarkers determination using commercial kits of Bio-diagnostic Co., Cairo, Egypt.

This supernatant was used for the determination of lipid peroxidation (LPO) according to the method of Ohkawa, et al. [58]. Briefly, the level of LPO was measured after incubation at 95 °C with thiobarbituric acid (TBA) in aerobic conditions (pH 3.4). The pink color produced by these reactions was measured spectrophotometrically at 532 nm. The activity of SOD was determined by the method described by Nishikimi, et al. [59]. The activity of SOD in liver and kidney tissues were measured calorimetrically based on the ability of SOD to inhibit the phenazine methosulphate-mediated reduction of nirtoblue tetrazolium dye. Estimation of CAT activity was conducted, where CAT reacts with a known quantity of H_2_O_2_. The reaction is stopped after exactly one minute with catalase inhibitor [60].

Reduced glutathione (GSH) was measured based on the reduction of 2-nirtobenzoic acid (DTNB) with glutathione (GSH) to produce a yellow compound. The reduced chromogen directly proportional to (GSH) concentration and its absorbance could be measured at 405 nm as described by Beutler [61]. Total antioxidant capacity (TAC) was assessed depend on the reaction with (±)-6-hydroxy-2,5,7,8-tetramethylchromane-2- carboxylic acid (Trolox) solution [62].

### 2.7. Statistical Analyses

All values from were presented as mean ± SD. Data obtained from the experiment were subjected to normality and homogeneity check to confirm the suitability for parametric test. One-way analysis of variance (ANOVA) test using SPSS v.19 (IBM SPSS Statistics for Windows, Version 19.0. Armonk, NY: IBM Corp.) was used to check the significant effects of treatments. Tukey’s test was used to differentiate the difference between means at *p* ≤ 0.05.

## 3. Results

### 3.1. Blood Performance

The blood performance analysis revealed a significant decrease in RBC, Hb, hematocrit, and other blood indices (MCV, MCH, and MCHC) with exposure to a sublethal dose of ABM (20.73 µg/L) (Table 2). Meanwhile, WBCs significantly increased in fish exposed to ABM. Dietary quercetin did not significantly improve the blood performance. However, quercetin supplementation to ABM-exposed fish succeeded in alleviating the negative impacts of ABM on blood performance indices in a dose-dependent manner.

### 3.2. Biochemical Parameters

The biochemical investigation of Nile tilapia fish exposed to ABM with or without quercetin dietary supplementation was presented in Table 3. The ABM exposure significantly decreased total protein, albumin, globulin, and significantly increased total lipids, cholesterol, and glucose content in fish serum compared to the control. Dietary supplementation of quercetin significantly increased total protein and globulin, but total lipids, cholesterol, and glucose were not significantly affected by dietary quercetin compared to the control. The co-supplementation of dietary quercetin to ABM-exposed fish restored the levels of different examined biochemical parameters, especially in groups supplemented with 800 mg quercetin/kg diet.

### 3.3. Liver and Kidney Functions

The hepatorenal functions in fish exposed to ABM and/or fed quercetin-supplemented diets were presented in Table 4. The activities of AST and ALT and the levels of urea and creatinine were significantly increased in serum of ABM-exposed fish compared to the control. Dietary quercetin did not significantly affect liver and kidney functions. Meanwhile, quercetin supplementation in the diet of ABM-exposed fish reduced the levels of AST, ALT, ALP, urea, and creatinine to be similar levels as in the control group, especially with the higher quercetin dose.

### 3.4. Antioxidant Status

Table 5 showed the antioxidant status in liver homogenate of Nile tilapia exposed to sublethal level of ABM and/or quercetin dietary supplementation. The LPO level was significantly increased in the liver homogenates of fish exposed to ABM toxicity. Meanwhile, fish fed quercetin-supplemented diets and exposed to ABM had significantly lower LPO compared to intoxicated fish. In addition, the antioxidant enzyme activities, including CAT, SOD, GSH, and TAC, were significantly decreased in ABM-exposed fish. The dietary supplementation of quercetin restored the activities of antioxidant enzymes and TAC levels in the ABM-exposed group in a dose-dependent manner to be similar to that reported in the control.

The effect of ABM exposure with or without quercetin dietary supplementation on antioxidant status in kidney tissue is shown in Table 6. The exposure to ABM significantly increased LPO and significantly decreased the activities of CAT, SOD, GSH, and TAC compared to the non-exposed group. Meanwhile, dietary quercetin significantly overcomes the effect of ABM on different antioxidant biomarkers.

### 3.5. Acetylcholinesterase Activity

**Figure 1:**
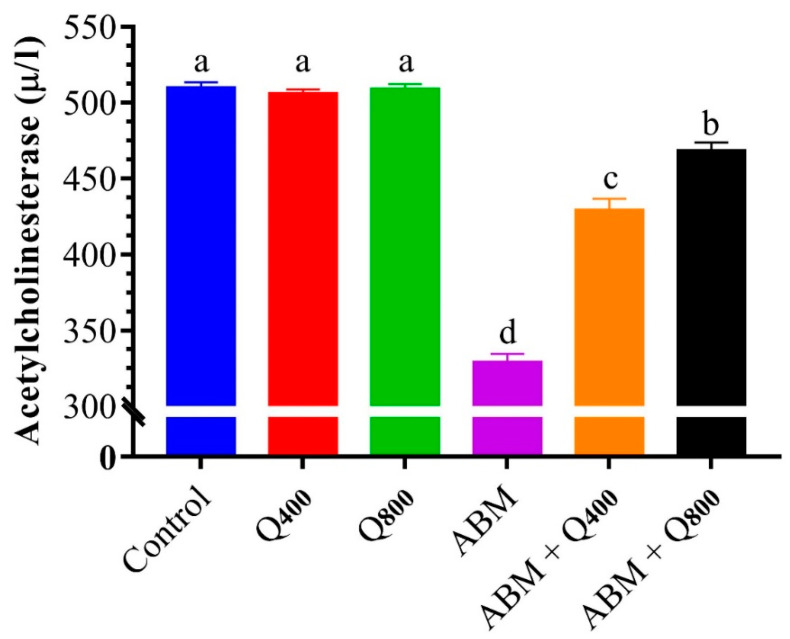
Effects of ABM exposure (20.73 µg/L) and dietary quercetin (400 and 800 mg/kg diet) on acetylcholinesterase of Nile tilapia, *Oreochromis niloticus*. Columns with different lowercase letters are significantly different (*p* ≤ 0.05) as analyzed by one-way ANOVA followed by Tukey’s post-hoc comparison. Q_400_: fish fed diet supplemented with 400 mg quercetin/kg diet, Q_800_: fish fed diet supplemented with 800 mg quercetin/kg diet, ABM: fish exposed to 20.73 µg ABM/L, ABM + Q_400_: fish fed diet supplemented with 400 mg quercetin/kg diet and exposed to 20.73 µg ABM/L, ABM + Q_800_: fish fed diet supplemented with 800 mg quercetin/kg diet and exposed to 20.73 µg ABM/L_,_ ABM.

The AchE activity significantly decreased in the fish exposed to ABM (Figure 1). Meanwhile, this enzyme activity was significantly increased with increasing quercetin levels in the ABM-exposed group. However, quercetin did not affect the AchE in normal groups.

## 4. Discussion

The obtained findings showed that exposure to a sublethal level of ABM caused severe anemia, as evidenced by a significant decrease in RBC count and volume and hemoglobin content and concentration. Our results agree with those of Hamed and El-Sayed [27], Fırat and Tutus [14], who documented a decrease in RBCs count and Hb content of Nile tilapia after 96 h exposure to 10 ppb ABM. Additionally, hybrid catfish exposed to 5 µ ABM/L for 20 days significantly decreased RBCs and Hb [13]. ABM showed negative blood performance changes and genotoxic effects, including abnormalities observed as erythrocytes with micronucleus, notched, eight-shaped, lobed nucleuses in African catfish, *C. gariepinus* [18]. Hematological status is the main bio-monitoring tool to judge fish health and assess the influence of pollutants [63,64]. The decline in RBCs count and Hb content with ABM exposure might be due to the pass of the insecticide into blood inducing low erythropoiesis and hemosynthesis dysfunction [65]. Conversely, the disruption of RBCs production and hemoglobin synthesis could be due to the inhibition effect of the toxic substances on the enzymatic systems involved in this process [66]. In addition, Al-Kahtani [67] reported a significant decrease in oxygen consumption rate associated with low blood hemoglobin in Nile tilapia exposed to ABM.

Meanwhile, the WBC count was significantly increased after exposure to ABM in the present study. The increase in WBC count can be interpreted as an indication of a chemical allergy. In the same line, hybrid catfish exposed to ABM experienced higher WBC counts [13]. Besides, it is well known that the increase of leucocytes is a typical response when fish are attacked by foreign substances [68].

The dietary supplementation of quercetin enhanced the blood performance via modulating hematological biomarkers that were negatively affected by ABM exposure. This improvement could be associated with the antioxidant effect of quercetin. Previous studies supported our outcomes and confirmed that quercetin had direct effects on blood and can act as a vasodilator, an antiplatelet, and has an antiproliferative impact, lessening oxidative damage and blood pressure [41,69,70]. In the present study, the blood performance in the control group were not significantly affected by quercetin. These results were in accordance with the findings of [41], who reported that quercetin did not affect the blood performance and biochemical parameters of silver catfish while improving antioxidant status.

Assessment of protein levels in fish blood is essential to indicate growth performance and tissue maintenance. It also stimulates the production of various components, such as enzymes, hormones, and antibodies [71]. In the present study, the exposure to ABM induced several blood biochemical disturbances, including hypoproteinemia, hypoalbuminemia, and hyperlipidemia. Our results agree with Kushwaha, et al. [16], who reported a significant decrease in serum proteins of *O. mossambicus* exposed to 45 and 55 ppb of ABM for 48 h. The decreased protein level may be due to alterations in the protein metabolism, whereas cell damage induced by ABM intoxication may affect the ability of cells to synthesize protein. In addition, protein may be utilized as a source of energy for repair of the damaged cells and compensate for stress induced-energy consumption [67,72,73,74]. The levels of some plasma immune components were significantly reduced in ABM-exposed common carp, including albumin, lysozyme, complement activity, and total immunoglobulin [19]. The decrease in blood proteins and related substances in the present study could be attributed to the impairment of protein synthesis in the liver or protein excretion by the kidney due to ABM-inducing liver and kidney failure [67]. Meanwhile, dietary quercetin improved the levels of plasma protein and globulin and restored the levels of different biochemical parameters in ABM-exposed fish. This could be attributed to the action of phenolic constituents found in quercetin in improving metabolism [75]. In accordance, dietary supplementation with curcumin and resveratrol alleviated the effect of ABM on the common carp plasma biochemical parameters [19].

ABM-exposed fish showed a significant increase in serum levels of total lipids and cholesterol levels. This could be a compensatory mechanism of Nile tilapia to cope with the ABM toxic stress. In accordance with our findings, Mahmoud, et al. [15] found that ABM altered the lipid profile via increasing the total cholesterol, triglycerides, low-density lipoprotein, and very low-density lipoprotein of Nile tilapia. On the other hand, supplementation of fish with quercetin ameliorated the increased levels of total lipids and cholesterol in the present study. In the same manner, dietary supplement of *S. chinensis* extract restored the level of total cholesterol to the normal in ABM-exposed Nile tilapia [15]. The antilipemic effect of quercetin could be attributed to the change of expression profiles of many lipid metabolism-related genes, such as *Fnta*, *Pparg*, *Aldh1b1*, *Pon1*, *Apoa4*, *Gpam*, *Abcg5*, *Acaca*, *Fdft1*, *Cd36*, and *Fasn*, and affected the lipogenesis process [76]. Additionally, quercetin may suppress lipogenesis and lipid deposition via regulating the cAMP signaling pathways [77]. In Nile tilapia, dietary quercetin up to 1600 mg/kg significantly reduced triglyceride and low-density lipoprotein cholesterol levels in the serum and whole fish body lipid content [38].

Glucose level in fish serum/plasma are an important indicator for the assessment of stress conditions caused by chemical toxicants [78,79,80]. ABM significantly increased the serum glucose level in serum of the exposed fish. Our results agree with those of Katharios, et al. [81], who recorded a significant increase in plasma glucose level of sea bream, *Sparus aurata,* intraperitoneally injected with 100, 200, 400, and 800 μg ivermectin/kg fish after 35 days of exposure. Fırat and Tutus [14] documented a significant increase in plasma glucose level after 96 h in Nile tilapia exposed to 10 ppb ABM and suggested that the glucose level was elevated because of the increased energy demand brought on by stress caused by ABM exposure, whereas higher glucose levels are important for the recovery from stress [79]. On the other hand, fish fed quercetin-supplemented diets and exposed to ABM had lower glucose levels. In accordance with the present findings, curcumin and resveratrol succeeded in mitigating the stress response induced by ABM by decreasing cortisol and glucose levels [19]. Furthermore, Yan, et al. [82] reported that quercetin can lower blood glucose levels via protecting pancreatic cells and/or improving insulin sensitivity.

Liver function enzymes are involved in several metabolic processes inside hepatocytes [83,84]. Their blood elevations reflect abnormal hepatic function and/or necrosis [85]. In the current study, the hepatotoxicity of ABM was indicated by the elevation of ALT and AST activities. Concurrent with recent reports, Fırat and Tutus [14] and Mahmoud, et al. [15] detected augmented levels of ALT and AST post-exposure of Nile tilapia to ABM. In addition, the exposure to ABM induced severe necrosis in the liver and degeneration of the tubular cell and glomerulus deformation in the kidney of hybrid catfish [13]. ALT and AST are essential enzymes in assessing hepatocellular damage and many hepatic disorders because they are sensitive responders to pollution [86]. The significant elevation in the activity of serum transaminases may be attributed to the degenerative changes in the hepatocytes and the hepato-oxidative damage necrosis resulting from hypoxia induced by ABM [16].

In addition, creatinine and urea concentrations were significantly increased in mice treated with ABM, which was associated with kidney histopathological changes [87]. Both creatinine and urea serum levels are important indicators of kidney function, whereas their elevation could be attributed to a reduction in glomerular filtration capacity and dysfunction of the kidney tubules [88]. On the other hand, fish fed a quercetin-enriched diet exhibited strong hepatoprotective activity via modulating levels of ALT and AST. Recent papers by Miltonprabu, et al. [89] and Ghafarifarsani, et al. [42] supported our finding and revealed that quercetin is a potential therapeutic medicinal agent for preventing hepatotoxicity through its efficient antioxidant and antiinflammatory action. Quercetin’s cell-protective activity may also be attributed to altering the activities of endogenous antioxidants to suppress apoptosis [90]. Concurrent with recent reports, Mahmoud, et al. [15] reported that the augmented levels of ALT and AST post-exposure of Nile tilapia to ABM restored by dietary supplementation of *Simmondsia chinensis* extract. The negative impact of ABM on liver integrity was mitigated by curcumin and resveratrol supplementation, whereas the activities of AST, ALP, and lactate dehydrogenase significantly decreased in the group exposed to ABM and fed different dietary supplements [19].

The antioxidant defense mechanism is among the immune responses that are responsible for protecting the fish body from oxidative damage [91]. The present findings showed an antioxidant imbalance in liver and kidney homogenates of fish exposed to a sublethal level of ABM. Moreover, a significant increase was found in the LPO level together with a significant decrease in the GSH content, TAC value, and SOD and CAT activities in liver and kidneys of ABM-exposed fish. This indicated the hepato-renal oxidative damage induced by ABM. This finding is consistent with the findings of Fırat and Tutus [14], who found that exposing Nile tilapia to 10 ppb ABM for 96 h caused a significant increase in MDA levels while decreasing GSH content and SOD, CAT, and glutathione reductase activities. The exposure to ABM reduced GSH and protein content in the liver and gills of *O. mossambica* [92]. In common carp, ABM exposure significantly decreased the activities of SOD and increased the level of MDA [19]. Mahmoud, et al. [15] reported a decline in the TAC and GSH content, and elevation in the MDA and nitric oxide levels in Nile tilapia exposed to sublethal dose of ABM. The ABM-induced oxidative damage may be attributed to its ability to increase the generation of ROS, which, when the capacity of antioxidants is exceeded, causes oxidative stress [93].

On the other hand, the current findings revealed that dietary supplementation of quercetin alleviated the negative effects of ABM on the antioxidant system, indicating potent antioxidant prosperities of quercetin. The functionality of quercetin in boosting the antioxidant response of Nile tilapia could be dominated by the suppression of the inflammatory species production (ROS, TNF, and IL-1) and enhancement of GSH levels and SOD [94]. In addition, quercetin upregulates the expression of Nrf2, which is a central regulator in the oxidative stress and a key factor for encoding antioxidant enzymes [95]. Additionally, quercetin upregulated SOD gene expressions, which was correlated to some extent to its higher activities [96]. The high phenolic compound content of quercetin, such as polyphenolic flavonoid compounds, has a powerful antioxidant and free radical scavenger [97]. In the same vein, dietary supplementation of curcumin and resveratrol alleviated the effect of ABM on antioxidant status [19]. *S. chinensis* extract-enriched diet alleviated the oxidative stress induced by a sublethal dose of ABM in Nile tilapia [15]. Dietary quercetin decreased LPO and thiobarbituric acid reactive substances and increased SOD, CAT, and glutathione S-transferase in silver catfish tissues [41]. In addition, the expression of Cu/Zn-SOD and CAT were upregulated in the liver of blunt snout bream fed a quercetin-supplemented diet [39]. Additionally, quercetin enhanced serum and liver antioxidant capacities of common carp [42].

Measurement of AchE activity is also a biomarker for pesticide exposure in several aquatic animals. In the current study, AchE activity was severely decreased with exposure to a sublethal level of ABM, which could be one of its modes of action as pesticides [98]. The effect of ABM on nervous systems could be due to the ability of this group of pesticides to pass through the blood–brain barrier of fish [99]. In accordance, a dose- and duration-dependent ivermectin exposure to *C. gariepinus* juvenile causes behavioral changes, abnormal mucus secretion, and skin color change, followed by spasms and paralysis [17]. Under controlled conditions, fish exposed to pesticides demonstrated suppression of AchE [100].

The dietary supplementation of quercetin ameliorated the effect of ABM on AchE. This improvement effect of quercetin could be due to its antioxidant activities, which in turn could stimulate immune cells in the nervous systems and induce antioxidant and antiinflammatory effects to maintain the integrity of the nervous systems [101]. In accordance with the present study, Bhattacharjee, et al. [102] reported that supplementation of freshwater teleost, *Channa punctata,* with quercetin ameliorated the AchE inhibitory effect induced by deltamethrin. Eventually, the current findings emphasized that quercetin had an antioxidant role in controlling blood performance, biochemical, hepato-renal, and antioxidant alterations induced by ABM exposure in Nile tilapia.

## 5. Conclusions

Based on the study outcomes, the abamectin (ABM) exposure induced significant negative blood performance changes, lower plasma protein profile, higher lipids, and cholesterol contents in Nile tilapia, *Oreochromis niloticus*. It also induced liver and kidney disfunction and oxidative damage. Dietary quercetin supplementation is a suitable strategy to boost Nile tilapia health status following exposure to ABM. Quercetin promotes hematopoiesis, blood protein, and GSH synthesis; consequently, the fish display lower stress and lipid peroxidation during the exposure to ABM. Such an enhancement in the antioxidant mechanism prevents oxidative damage to the fish liver and kidney. According to the present findings, dietary 800 mg/kg diet quercetin supplementation for a 60-day period is adequate to support the fish in ameliorating ABM toxicity.

## Figures and Tables

**Table 1:** Ingredients and proximate chemical analysis (%; on dry matter basis) of experimental diets containing different levels of quercetin.

Ingredients	Quercetin Levels (g/kg Diet)		
	0	0.4	0.8
Fish meal (72% crude protein)	85	85	85
Soybean meal (45% crude protein)	465	465	465
Wheat bran	183	183	183
Yellow corn	100	100	100
Corn oil	20	20	20
Cod liver oil	20	20	20
Mineral mixture ^a^	30	30	30
Vitamin mixture ^b^	30	30	30
Starch	67	66.6	66.2
Quercetin	0	0.4	0.8
Chemical composition (g/kg)			
Dry matter	915		
Crude protein	307		
Total lipids	91		
Crude fiber	48		
Total ash	61		
Nitrogen free extract (NFE) ^c^	493		
Gross energy (kcal/kg diet) ^d^	4814		

^a^ Vitamin premix (per kg of premix): thiamine, 2.5 g; riboflavin, 2.5 g; pyridoxine, 2.0 g; inositol, 100.0 g; biotin, 0.3 g; pantothenic acid, 100.0 g; folic acid, 0.75 g; paraaminobenzoic acid, 2.5 g; choline, 200.0 g; nicotinic acid, 10.0 g; cyanocobalamine, 0.005 g; a-tocopherol acetate, 20.1 g; menadione, 2.0 g; retinol palmitate, 100,000 IU; cholecalciferol, 500,000 IU. ^b^ Mineral premix (g/kg of premix): CaHPO_4_·2H_2_O, 727.2; MgCO_4_·_7_H_2_O, 127.5; KCl 50.0; NaCl, 60.0; FeC_6_H_5_O_7_·3H_2_O, 25.0; ZnCO_3_, 5.5; MnCl_2_·4H_2_O, 2.5; Cu(OAc)_2_·H_2_O, 0.785; CoCl_3_·6H2O, 0.477; CaIO_3_·6H_2_O, 0.295; CrCl_3_·6H_2_O, 0.128; AlCl_3_·6H_2_O, 0.54; Na_2_SeO_3_, 0.03. ^c^ Nitrogen free extract = 100 − (protein % + lipid % + ash % + crude fiber %). ^d^ Gross energy: Calculated after NRC (2011) as 5.64, 9.44, and 4.11 Kcal/g for protein, lipid, and NFE, respectively.

**Table 2:** Effect of abamectin (ABM) exposure (20.73 µg/L) and dietary quercetin (400 and 800 mg/kg diet) on blood performance blood of Nile tilapia, *Oreochromis niloticus*.

Items	Red Blood Cells (10^6^/mm^3^)	White Blood Cells (10^3^/mm^3^)	Hemoglobin (g/dL)	Hematocrit (%)	Mean Cell Volume (fL)	Mean Corpuscular Hemoglobin (Pg/cell)	Mean Corpuscular Hemoglobin Concentrate (g/dL)
Control	3.74 ± 0.07 ^a^	0.99 ± 0.05 ^c^	7.52 ± 0.57 ^a^	32.71 ± 1.46 ^a^	110.57 ± 3.21 ^a^	29.71 ± 0.97 ^a^	21.23 ± 0.67 ^a^
Q_400_	3.71 ± 0.22 ^ab^	0.96 ± 0.04 ^d^	7.36 ± 0.86 ^a^	32.86 ± 1.53 ^a^	106.7 ± 2.61 ^b^	28.42 ± 1.03 ^ab^	20.59 ± 0.16 ^a^
Q_800_	3.79 ± 0.13 ^a^	0.95 ± 0.12 ^d^	7.43 ± 0.29 ^a^	31.29 ± 1.15 ^b^	108.42 ± 2.72 ^a^	28.12 ± 1.18 ^ab^	21.12 ± 0.12 ^a^
ABM	1.99 ± 0.05 ^d^	2.64 ± 0.32 ^a^	4.22 ± 0.15 ^c^	20.80 ± 1.17 ^d^	90.80 ± 1.25 ^d^	17.87 ± 0.75 ^c^	12.07 ± 0.15 ^c^
ABM + Q_400_	2.87 ± 0.35 ^c^	1.97 ± 0.24 ^b^	6.24 ± 0.41 ^b^	29.31 ± 1.72 ^c^	101.24 ± 2.16 ^c^	24.45 ± 1.26 ^b^	18.24 ± 1.46 ^b^
ABM + Q_800_	3.11 ± 0.22 ^b^	0.97 ± 0.06 ^c^	7.01 ± 0.52 ^a^	32.89 ± 1.68 ^a^	109.22 ± 3.11 ^a^	29.34 ± 1.19 ^a^	21.8 ± 1.85 ^a^

Means with different superscript are significantly different (*p* ≤ 0.05) as analyzed by one-way ANOVA followed by Tukey’s post-hoc comparison. Q_400_: fish fed diet supplemented with 400 mg quercetin/kg diet, Q_800_: fish fed diet supplemented with 800 mg quercetin/kg diet, ABM: fish exposed to 20.73 µg ABM/L, ABM + Q_400_: fish fed diet supplemented with 400 mg quercetin/kg diet and exposed to 20.73 µg ABM/L, ABM + Q_800_: fish fed diet supplemented with 800 mg quercetin/kg diet and exposed to 20.73 µg ABM/L.

**Table 3:** Effect of ABM exposure (20.73 µg/L) and dietary quercetin (400 and 800 mg/kg diet) on blood biochemical parameters of Nile tilapia, *Oreochromis niloticus*.

Items	Total Protein (g/dL)	Albumin (g/dL)	Globulin (g/dL)	Total Lipids (g/dL)	Cholesterol (g/dL)	Glucose (mg/dL)
Control	5.22 ± 0.67 ^c^	3.64 ± 0.08 ^a^	1.58 ± 0.27 ^d^	33.14 ± 2.18 ^c^	41.08 ± 3.57 ^c^	52.02 ± 1.27 ^bc^
Q_400_	6.25 ± 0.78 ^a^	3.39 ± 0.16 ^bc^	2.86 ± 0.14 ^b^	35.09 ± 2.23 ^c^	43.26 ± 3.18 ^c^	51.36 ± 1.34 ^c^
Q_800_	6.56 ± 0.41 ^a^	3.14 ± 0.12 ^c^	3.42 ± 0.20 ^a^	36.04 ± 3.27 ^c^	45.40 ± 3.05 ^c^	52.47 ± 1.80 ^bc^
ABM	3.11 ± 0.13 ^d^	1.43 ± 0.09 ^d^	1.68 ± 0.25 ^c^	51.03 ± 2.69 ^a^	71.50 ± 3.10 ^a^	70.12 ± 0.86 ^a^
ABM + Q_400_	5.89 ± 0.35 ^b^	3.45 ± 0.10 ^b^	2.44 ± 0.16 ^bc^	40.85 ± 2.39 ^b^	56.42 ± 3.75 ^b^	55.52 ± 0.98 ^b^
ABM + Q_800_	5.20 ± 0.48 ^c^	3.60 ± 0.03 ^a^	1.60 ± 0.23 ^d^	32.80 ± 3.05 ^c^	42.21 ± 3.46 ^c^	49.98 ± 1.54 ^c^

Means with different superscript are significantly different (*p* ≤ 0.05) as analyzed by one-way ANOVA followed by Tukey’s post-hoc comparison. Q_400_: fish fed diet supplemented with 400 mg quercetin/kg diet, Q_800_: fish fed diet supplemented with 800 mg quercetin/kg diet, ABM: fish exposed to 20.73 µg ABM/L, ABM + Q_400_: fish fed diet supplemented with 400 mg quercetin/kg diet and exposed to 20.73 µg ABM/L, ABM + Q_800_: fish fed diet supplemented with 800 mg quercetin/kg diet and exposed to 20.73 µg ABM/L.

**Table 4:** Effect of ABM exposure (20.73 µg/L) and dietary quercetin (400 and 800 mg/kg diet) on liver and kidney function parameters of Nile tilapia, *Oreochromis niloticus*.

Items	AST (μ/L)	ALT (μ/L)	Urea (mg/dL)	Creatinine (mg/dL)
Control	22.17 ± 0.89 ^c^	63.26 ± 8.28 ^c^	19.22 ± 1.47 ^c^	0.25 ± 0.04 ^c^
Q_400_	25.60 ± 2.79 ^c^	63.56 ± 3.48 ^c^	21.25 ± 1.28 ^c^	0.27 ± 0.08 ^c^
Q_800_	24.43 ± 1.21 ^c^	62.82 ± 3.60 ^c^	22.36 ± 1.61 ^c^	0.27 ± 0.05 ^c^
ABM	73.82 ± 2.45 ^a^	129.43 ± 6.75 ^a^	35.43 ± 2.13 ^a^	0.80 ± 0.09 ^a^
ABM + Q_400_	35.11 ± 5.62 ^b^	94.77 ± 5.20 ^b^	25.19 ± 0.44 ^b^	0.42 ± 0.02 ^b^
ABM + Q_800_	23.28 ± 1.72 ^c^	62.29 ± 3.72 ^c^	18.43 ± 1.25 ^c^	0.24 ± 0.01 ^c^

Means with different superscript are significantly different (*p* ≤ 0.05) as analyzed by one-way ANOVA followed by Tukey’s post-hoc comparison. Q_400_: fish fed diet supplemented with 400 mg quercetin/kg diet, Q_800_: fish fed diet supplemented with 800 mg quercetin/kg diet, ABM: fish exposed to 20.73 µg ABM/L, ABM + Q_400_: fish fed diet supplemented with 400 mg quercetin/kg diet and exposed to 20.73 µg ABM/L, ABM + Q_800_: fish fed diet supplemented with 800 mg quercetin/kg diet and exposed to 20.73 µg ABM/L.

**Table 5:** Effect of ABM exposure (20.73 µg/L) and dietary quercetin (400 and 800 mg/kg diet) on antioxidant profile in liver homogenate of Nile tilapia, *Oreochromis niloticus*.

Items	Lipid Peroxidation (nmol/mg)	Catalase (Ug/mg)	Super Oxide Dismutase (Ug/mg)	Reduced Glutathione (nmol/mg)	Total Antioxidant Capacity (Umol/mg)
Control	63.29 ± 2.58 ^c^	25.24 ± 2.25 ^a^	12.06 ± 0.74 ^a^	35.85 ± 2.57 ^a^	3.99 ± 0.12 ^a^
Q_400_	60.43 ± 2.52 ^c^	24.32 ± 2.31 ^ab^	11.42 ± 0.69 ^ab^	34.16 ± 2.24 ^a^	3.15 ± 0.14 ^ab^
Q_800_	64.38 ± 3.19 ^c^	24.84 ± 2.41 ^ab^	12.11 ± 0.51 ^a^	35.06 ± 1.86 ^a^	3.44 ± 0.13 ^a^
ABM	86.10 ± 2.30 ^a^	11.81 ± 0.60 ^c^	7.34 ± 0.15 ^c^	15.92 ± 0.49 ^c^	0.99 ± 0.54 ^d^
ABM + Q_400_	69.22 ± 3.01 ^b^	18.35 ± 0.12 ^c^	10.68 ± 0.26 ^b^	29.91 ± 1.63 ^b^	2.85 ± 0.31 ^c^
ABM + Q_800_	60.10 ± 3.13 ^c^	20.99 ± 1.16 ^b^	12.25 ± 0.60 ^a^	32.48 ± 1.63 ^ab^	3.02 ± 0.33 ^ab^

Means with different superscript are significantly different (*p* ≤ 0.05) as analyzed by one-way ANOVA followed by Tukey’s post-hoc comparison. Q_400_: fish fed diet supplemented with 400 mg quercetin/kg diet, Q_800_: fish fed diet supplemented with 800 mg quercetin/kg diet, ABM: fish exposed to 20.73 µg ABM/L, ABM + Q_400_: fish fed diet supplemented with 400 mg quercetin/kg diet and exposed to 20.73 µg ABM/L, ABM + Q_800_: fish fed diet supplemented with 800 mg quercetin/kg diet and exposed to 20.73 µg ABM/L.

**Table 6:** Effects of ABM exposure (20.73 µg/L) and dietary quercetin (400 and 800 mg/kg diet) on antioxidant profile in kidney homogenate of Nile tilapia, *Oreochromis niloticus*.

Items	Lipid Peroxidation (nmol/mg)	Catalase (Ug/mg)	Super Oxide Dismutase (Ug/mg)	Reduced Glutathione (nmol/mg)	Total Antioxidant Capacity (Umol/mg)
Control	72.09 ± 1.96 ^c^	16.21 ± 1.21 ^b^	15.74 ± 2.09 ^a^	28.65 ± 3.27 ^a^	12.21 ± 0.43 ^a^
Q_400_	75.40 ± 1.69 ^c^	18.02 ± 1.34 ^a^	15.08 ± 2.74 ^a^	27.03 ± 3.05 ^a^	12.19 ± 0.35 ^a^
Q_800_	75.30 ± 2.08 ^c^	18.51 ± 1.41 ^a^	15.55 ± 1.98 ^a^	26.58 ± 3.90 ^a^	13.65 ± 0.63 ^a^
ABM	98.23 ± 2.53 ^a^	8.26 ± 0.90 ^d^	9.47 ± 1.78 ^c^	17.02 ± 0.32 ^c^	5.53 ± 0.09 ^c^
ABM + Q_400_	80.29 ± 1.87 ^b^	13.28 ± 0.29 ^c^	12.34 ± 1.46 ^b^	22.67 ± 2.07 ^b^	9.41 ± 0.36 ^b^
ABM + Q_800_	71.29 ± 2.61 ^d^	16.30 ± 1.16 ^b^	15.01 ± 1.31 ^a^	26.05 ± 3.17 ^a^	11.18 ± 0.17 ^a^

Means with different superscript are significantly different (*p* ≤ 0.05) as analyzed by one-way ANOVA followed by Tukey’s post-hoc comparison. Q_400_: fish fed diet supplemented with 400 mg quercetin/kg diet, Q_800_: fish fed diet supplemented with 800 mg quercetin/kg diet, ABM: fish exposed to 20.73 µg ABM/L, ABM + Q_400_: fish fed diet supplemented with 400 mg quercetin/kg diet and exposed to 20.73 µg ABM/L, ABM + Q_800_: fish fed diet supplemented with 800 mg quercetin/kg diet and exposed to 20.73 µg ABM/L.

## Data Availability

Data are available upon reasonable request.
